# The *Arabidopsis* adaptor protein AP-3µ interacts with the G-protein β subunit AGB1 and is involved in abscisic acid regulation of germination and post-germination development

**DOI:** 10.1093/jxb/ert327

**Published:** 2013-10-05

**Authors:** Jeeraporn Kansup, Daisuke Tsugama, Shenkui Liu, Tetsuo Takano

**Affiliations:** ^1^Asian Natural Environmental Science Center, The University of Tokyo, Nishitokyo, Tokyo 188-0002, Japan; ^2^Laboratory of Plant Molecular Genetics, Graduate School of Agricultural and Life Sciences, University of Tokyo, 1-1-1 Yayoi, Bunkyo-ku, Tokyo 113–8657, Japan; ^3^Alkali Soil Natural Environmental Science Center, Northeast Forestry University, Harbin 150040, China

**Keywords:** Abscisic acid, *Arabidopsis*, G-protein, adaptor protein, germination, post-germination growth.

## Abstract

Heterotrimeric G-proteins (G-proteins) have been implicated in ubiquitous signalling mechanisms in eukaryotes. In plants, G-proteins modulate hormonal and stress responses and regulate diverse developmental processes. However, the molecular mechanisms of their functions are largely unknown. A yeast two-hybrid screen was performed to identify interacting partners of the *Arabidopsis* G-protein β subunit AGB1. One of the identified AGB1-interacting proteins is the *Arabidopsis* adaptor protein AP-3µ. The interaction between AGB1 and AP-3µ was confirmed by an *in vitro* pull-down assay and bimolecular fluorescence complementation assay. Two *ap-3µ* T-DNA insertional mutants were found to be hyposensitive to abscisic acid (ABA) during germination and post-germination growth, whereas *agb1* mutants were hypersensitive to ABA. During seed germination, *agb1*/*ap-3µ* double mutants were more sensitive to ABA than the wild type but less sensitive than *agb1* mutants. However, in post-germination growth, the double mutants were as sensitive to ABA as *agb1* mutants. These data suggest that AP-3µ positively regulates the ABA responses independently of AGB1 in seed germination, while AP-3µ does require AGB1 to regulate ABA responses during post-germination growth.

## Introduction

Heterotrimeric G-proteins (G-proteins) are conserved among eukaryotes and are responsible for the transmission of extracellular signals perceived by G-protein-coupled receptors to intracellular effectors. G-proteins consist of three subunits, Gα, Gβ, and Gγ. The *Arabidopsis thaliana* genome encodes one Gα (GPA1) (Ma *et al.*, 1990), one Gβ (AGB1) (Weiss *et al.*, 1994), and three Gγs (AGG1, AGG2, and AGG3) (Mason and Botella, 2000, 2001; Chakravorty *et al.*, 2011).

Studies on loss-of-function alleles and gain-of-function overexpression lines of G-protein subunits suggest that the G-proteins modulate hormonal and stress responses, and play regulatory roles in many growth and developmental processes (reviewed by Jones and Assmann, 2004; Chen, 2008). *agb1* mutants are characterized by aberrant leaf and flower shape, increased production of lateral root primordial, and shorter hypocotyls and siliques (Lease *et al.*, 2001; Ullah *et al.*, 2001, 2003). Additionally, seed germination and early seedling development of *agb1* mutants are hypersensitive to abscisic acid (ABA). Because plants lacking AGB1 have greater ABA hypersensitivity than plants lacking GPA1, AGB1 has been suggested to be the predominant regulator of G-protein-mediated ABA signalling (Pandey *et al.*, 2006). ABA was shown to be bound by GTG1 and GTG2, which are Gα-interacting receptors on the plasma membrane (Pandey *et al.*, 2009). A quantitative proteomics-based analysis of WT and *gtg1gtg2* mutants revealed that the majority of ABA-responsive proteins require the presence of GTG proteins (Alvarez *et al.*, 2013), supporting the importance of the G-proteins in ABA signal transduction.

The multiple phenotypes of *agb1* mutants suggest that AGB1 is a key factor of several signalling pathways. So far some genetic and/or physical AGB1-interaction partners have been identified and characterized, for example a Golgi-localized hexose transporter SGB1 (Wang *et al.*, 2006), an N-MYC downregulated-like1 (NDL1) (Mudgil *et al.*, 2009), and an acireductone dioxygenase-like protein, ARD1 (Friedman *et al.*, 2011). An interactome analysis revealed the involvement of G-proteins in cell wall modification (Klopffleisch *et al.*, 2011). However, the molecular mechanisms underlying the AGB1-mediated signalling are unclear (Klopffleisch *et al.*, 2011).

To identify interacting partners of AGB1, we performed a yeast two-hybrid screen (Kobayashi *et al.*, 2012; Tsugama *et al.*, 2012a). One of the AGB1-interacting proteins found in the screen was an adaptor protein, AP-3µ (At1g56590). Adaptor proteins (APs) are key regulators of endocytosis and secretory pathways. Five different heterotetrameric AP complexes (AP-1, AP-2, AP-3, AP-4, and AP-5) have been characterized so far in eukaryotes. The AP-3 complex, which consists of two large subunits (δ and β3), a medium subunit (µ3), and a small subunit (σ3) (Boehm and Bonifacino, 2002; Dell’Angelica, 2009), participates in protein sorting at the trans-Golgi network and/or endosome (Cowles *et al.*, 1997; Dell’Angelica *et al.*, 1997; Stepp *et al.*, 1997; Kretzschmar *et al.*, 2000).

In *Arabidopsis*, each subunit of the AP-3 complex is encoded by a single-copy gene (Bassham *et al.*, 2008). Loss-of function mutants of several subunits of the AP-3 complex have been shown to be the suppressors of *zigzag1* (*zig1*), which is abnormal in both shoot gravitropism and morphology due to the lack of a vesicle trafficking regulator, SNARE VTI11 (Niihama *et al.*, 2009). The AP-3 complex also plays a role in vacuolar function in *Arabidopsis*, including mediation of the transition between storage and lytic vacuolar identity (Feraru *et al.*, 2010; Zwiewka *et al.*, 2011). However, it is unclear whether the AP-3 complex also has roles in stress and hormonal responses.

Here we show that AP-3µ physically interacts with AGB1 in yeast and *in vitro*, as well as *in planta*. Genetic interaction between *AP-3µ* and *AGB1* is also examined using *agb1*/*ap-3µ* double mutants.

## Materials and methods

### Plant material and culture conditions

*A. thaliana* ecotype Columbia-0 (Col-0) was used throughout the experiments. Seeds of *ap-3µ-2* (Niihama *et al.*, 2009), *ap-3µ-4*, *agb1-1* (Lease *et al.*, 2001), *agb1-2* (Ullah *et al.*, 2003), *ap-3δ*, and *chc1-2* (Kitakura *et al.*, 2011) mutants were obtained from the *Arabidopsis* Biological Research Center (ABRC) with stock numbers of SALK_064486C, CS859652, CS3976, CS6536, SALK_144344C, and CS25142, respectively. The genetic backgrounds for all the mutant lines are Col-0. Except *agb1-1* mutant, T-DNA insertion was confirmed by genomic PCR analysis (Supplementary Table S1, available at *JXB* online). Seeds were surface sterilized and sown on 0.8% agar containing 0.5×Murashige and Skoog (MS) salts (Wako, Japan), 1% (w/v) sucrose, and 0.5g/l MES pH 5.8, with 0, 0.25, 0.5, 1.0, or 2.0 µM ABA or 400mM mannitol or 150mM NaCl or 9.2% polyethylene glycol, chilled at 4 °C in the dark for 3 d (stratified), and germinated at 22 °C. Plants were grown at 22 °C under 16/8 light/dark conditions.

### Yeast two-hybrid analysis

A yeast two-hybrid screen using AGB1 as a bait was performed as described previously (Tsugama *et al.*, 2012a). The construct of pGAD-AP-3µ was generated as described in Supplementary Method S1.

To confirm the result of the yeast two-hybrid screen, pGBK-AGB1 and pGAD-AP-3µ were co-introduced into the *Saccharomyces cerevisiae* strain AH109. After transformation, at least four colonies grown on SD media lacking leucine and tryptophan (SD/–Leu/–Trp), were streaked on SD/–Leu/–Trp and SD media lacking leucine, tryptophan, and histidine (SD/–Leu/–Trp/–His).

### *In vitro* pull-down assay

Polyhistidine-tagged AGB1 (His-AGB1) and polyhistidine-tagged AGG1 (His-AGG1) were expressed in *Escherichia coli* and purified as previously described (Tsugama *et al.*, 2012a). The constructs of pGEX-5X-AP-3µ and pGEX-5X- AP-3µ^DN^, which express GST-fused AP-3µ (GST-AP-3µ) and GST-fused AP-3µ^DN^ (GST-AP-3µ^DN^) respectively, were generated as described in Supplementary Method S2.

GST-AP-3µ and GST-AP-3µ^DN^ were induced and purified as described in Supplementary Method S3. GST-AP-3µ or GST-AP-3µ^DN^ in the crude extracts was bound to Glutathione Sepharose 4 Fast Flow (GE Healthcare, UK) following the manufacturer’s instructions, and the resin was washed four times with phosphate-buffered saline (PBS, 137mM NaCl, 8.10mM Na_2_HPO_4_.12H_2_O, 2.68mM KCl, 1.47mM KH_2_PO_4_, pH 7.4). After removing the PBS, the resin was resuspended in solution containing purified His-AGB1 and incubated at room temperature for 60min with gentle shaking. The resin was then washed four times with PBS and resuspended in 20mM reduced glutathione in 50mM Tris-HCl pH 8.0. The suspension was incubated at room temperature for 15min to release GST-AP-3µ or GST-AP-3µ^DN^. The slurry of the resin was centrifuged for a few minutes at 12 000 *g*. GST-AP-3µ or GST-AP-3µ^DN^ and His-AGB1 in the supernatant were analysed by immunoblotting using an anti-GST antibody (diluted 4000-fold; GE Healthcare, UK) and HisProbe-horseradish peroxidase (HRP) (diluted 2000-fold; Thermo Fisher Scientific, USA). After the reaction of an anti-GST antibody, HRP-linked rabbit antibodies against goat IgG (diluted 5000-fold; MBL, Japan) were used as second antibodies. Signals were detected with SuperSignal West Pico Chemiluminescent Substrate (Thermo Fisher Scientific).

### Bimolecular fluorescence complementation assay

To express cYFP (the C-terminal half of YFP, yellow fluorescence protein)-fused AP-3µ, the open reading frame (ORF) of *AP-3µ* was amplified by PCR using pGAD-AP-3µ as template and the following primer pair: 5′-CCGGTCTAGAATGCTTCAATGTATCTTTCTC-3′ and 5′-GGCGCCCGGGTACAACCTGACATCGAACTCACCAGC-3′ (*Xba*I and *Sma*I sites underlined). The PCR products were cloned into the *Sma*I site of pBluescript II SK^–^. The resultant plasmid was digested by *Xba*I, and the resultant ORF fragments of AP-3µ were inserted into the *Spe*I site of pBS-35SMCS-cYFP (Tsugama *et al.*, 2012a), generating pBS-35S-AP-3µ-cYFP. To express nYFP (the N-terminal half of YFP)-fused AGB1, pBS-35S-nYFP-AGB1 (Tsugama *et al.*, 2012a) was used. A mixture of an nYFP construct and a cYFP construct (500ng each) was used for particle bombardment to co-express proteins of interest in onion epidermal cells. Particle bombardment and fluorescence microscopy were performed as previously described (Zhang *et al.*, 2008). Images were processed using Canvas X software (ACD Systems).

### Subcellular localizations of GFP- and mCherry-fused proteins

The constructs of pBI121-35S-GFP, pBI121-35S-AP-3µ-GFP, pBI121-35S-mCherry, and pBI121-35S-AGB1-mCherry were generated as described in Supplementary Method S4. A mixture of pBI121-35S-AP-3µ-GFP and pBI121-35S-AGB1-mCherry (1 µg each) or pBI121-35S-GFP and pBI121-35S-mCherry (for control) was used for particle bombardment to co-express AP-3µ-GFP and AGB1-mCherry or GFP alone and mCherry alone in onion epidermal cells. Particle bombardment and fluorescence microscopy were performed as previously described (Zhang *et al.*, 2008). For ABA treatment, the bombarded onion epidermal cells were incubated in 0.5×MS containing 100 µM ABA for 1h before microscopy observation. Images were processed using Canvas X software.

### Measurement of germination and greening rates

Germination and greening rates were compared between seed lots that were produced, harvested, and stored under identical conditions. Seeds were sown and grown as already described. Germination was defined here as emergence of the radicle through the seed coat. Cotyledon greening was defined as obvious cotyledon expansion and turning green. Germination and greening rates were scored daily for 9 days after seeds were transferred to the light at 22 °C. The experiments were repeated at least twice. The data shown are the means of all the experiments ± SD.

### Semi-quantitative and quantitative real-time reverse-transcription PCR

The expression of *AP-3µ* mRNA in the wild type and the *ap-3µ* mutants was tested by semi-quantitative reverse-transcription (RT) PCR. Plants of each genotype were grown for 2 weeks and sampled. Total RNA was prepared using the GTC method (Chomczynski and Sacchi, 1987) and cDNA was synthesized from 4.6 µg of total RNA with PrimeScript Reverse Transcriptase (Takara, Japan) using an oligo(dT) primer. The primers used for the RT-PCR are shown in Supplementary Fig. S1A and the primer sequences are given in Supplementary Table S2. The expressions of the ABA-responsive genes (*RAB18*, *RD29A*, and *AHG1*) in the wild type and the *ap-3µ* mutants were tested by quantitative real-time RT-PCR. Plants of each genotype were grown for 18 days on 0.8% agar containing 0.5×MS salts 1% (w/v) sucrose, and 0.5g/l MES, pH 5.8, with 0 or 1.0 µM ABA and sampled. Total RNA was prepared using RNeasy Plant Mini Kit (Qiagen, Netherlands) and cDNA was synthesized from 2 µg of the total RNA with High Capacity RNA-to-cDNA Kit (Applied Biosystems, USA) according to the manufacturer’s instructions. The reaction mixtures were diluted 20 times with distilled water and used as a template for PCR. The primer sequences are given in Supplementary Table S2 (Nishimura *et al.*, 2007; Tsugama *et al.*, 2012b). Quantitative real-time RT-PCR was performed using SYBR Premix Ex Taq II (Perfect Real Time) (Takara) and the StepOne Real-Time PCR System (Applied Biosystems).

## Results

### AP-3µ interacts with AGB1

**Fig. 1.**
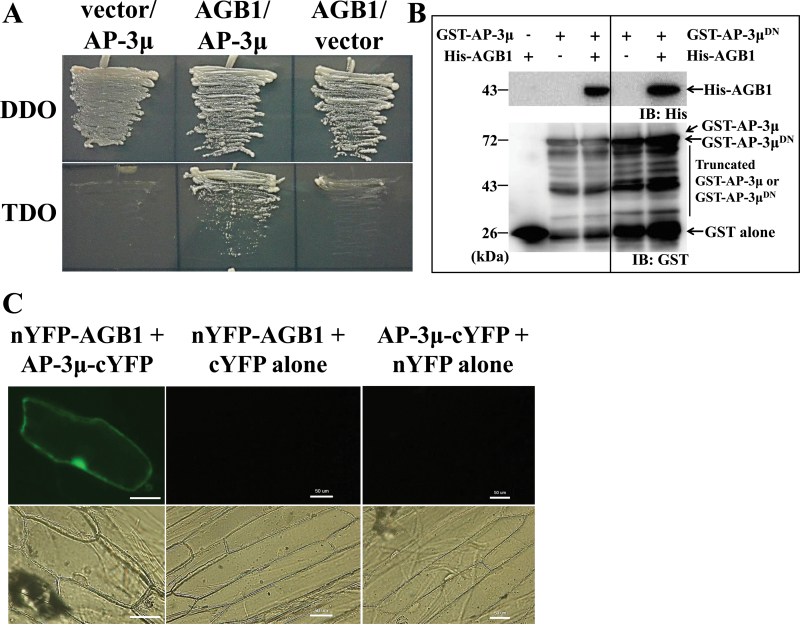
Interaction between AP-3µ and AGB1. (A) Yeast two-hybrid assay. The combinations of the plasmids used for transformation of the yeast strain AH109 are indicated at the top of the panel. vector, a pGBKT7 plasmid or a pGADT7-Rec plasmid containing no insert; AGB1, pGBKT7-AGB1; AP-3µ, pGADT7-Rec-AP-3µ (full-length ORF). Yeast cells were cultured on DDO (SD/–Trp/–Leu or control) and TDO (SD/–Trp/–Leu/–His) plates to check activation of the reporter gene, HIS3. At least four colonies were tested and a representative result is shown. (B) *In vitro* GST pull-down assay. GST-fused AP-3µ (GST-AP-3µ) or GST-fused AP-3µ^DN^ (GST-AP-3µ^DN^), respectively and His-tagged AGB1 (His-AGB1) were expressed in *Escherichia coli* and used for the analysis. The presence or absence of each protein in the reaction mixture is shown as + or –, respectively. Experiments were performed four times and a representative result is shown. Antibodies used for immunoblotting are shown as IB:His and IB:GST. (C) Bimolecular fluorescence complementation in onion epidermal cells. The ORF of AGB1 was cloned in frame behind the coding sequence of the N-terminal region of YFP (nYFP) to express nYFP-fused AGB1 (nYFP-AGB1), and the ORF of AP-3µ was cloned in frame in front of the coding sequence of the C-terminal region of YFP (cYFP) to express cYFP-fused AP-3µ (AP-3µ-cYFP). Both constructs were introduced into onion epidermal cells. cYFP alone and nYFP alone were used as controls. More than 20 cells were observed and a representative cell is shown. Bars=50 µm (this figure is available in colour at *JXB* online).

To identify interacting partners of AGB1, we performed a yeast two-hybrid screen of the *Arabidopsis* leaf library using full-length AGB1 as bait. Even on high-stringency selection media (SD/QDO), more than 3600 positive clones were obtained. Using yeast colony PCR with an *AGG1*- or *AGG2*-specific primer, we found that 60–70% of these clones expressed AGG1 (data not shown). Plasmid inserts from non-AGG1 clones were then amplified by colony PCR using a vector-specific primer pair, and sequenced. Around 400 clones were sequenced, and three of them expressed adaptor protein AP-3µ (GenBank accession: BX814222; At1g56590). Yeast cells could grow on the selection medium when both AP-3µ and AGB1 were present, but not when either of them was absent, indicating that AP-3µ and AGB1 interact with each other in yeast cells (Fig. 1A).

To confirm that AP-3µ binds directly to AGB1, we studied their interactions using a GST pull-down assay. His-AGB1 was detected only when it was reacted with AP-3µ, indicating that AGB1 and AP-3µ interact *in vitro* (Fig. 1B). Although the C-terminal 18 amino acids of AP-3µ are needed for recruiting cargo into the forming vesicle (Owen and Evans, 1998), they are not needed for the interaction with AGB1 because His-AGB1 was also detected when it was reacted with AP-3µ^DN^, which lacks the C-terminal 18 amino acids (Fig. 1B right).

In a bimolecular fluorescence complementation assay, YFP fluorescence was recovered in the cytosol and the nucleus when nYFP-AGB1 (AGB1 fused to the N-terminal half of YFP) and AP-3µ-cYFP (AP-3µ fused to the C-terminal half of YFP) were coexpressed (Fig. 1C), suggesting that they interact in the cytosol and nucleus in plant cells.

### Subcellular localizations of AP-3µ and AGB1

**Fig. 2.**
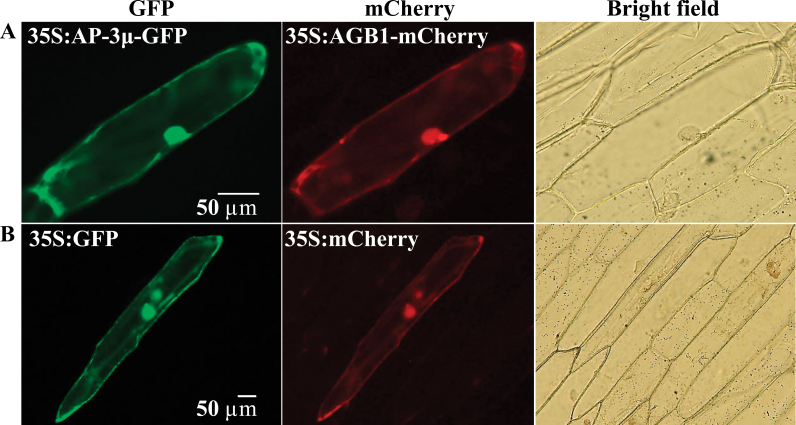
Subcellular localizations of AP-3µ and AGB1. GFP-fused AP-3µ (AP-3µ-GFP) and mCherry-fused AGB1 (AGB1-mCherry) (A) or GFP alone and mCherry alone (B) were transiently co-expressed in onion epidermal cells under the control of 35S promoter. More than 10 cells were observed and a representative cell is shown in each panel. Bars=50 µm (this figure is available in colour at *JXB* online).

When co-expressed in onion epidermal cells, GFP-fused AP-3µ (AP-3µ-GFP) was detected in the cytoplasm and nucleus, while mCherry-fused AGB1 (AGB1-mCherry) was detected in the cytoplasm, nucleus, and the plasma membrane (Fig. 2), suggesting the possibility that AP-3µ and AGB1 are co-localized in the cytoplasm and nucleus. This result is consistent with the above-described cytoplasmic and nuclear bimolecular fluorescence complementation between AP-3µ and AGB1 (Fig. 1C). ABA treatment did not affect the patterns of signals of either AP-3µ-GFP or AGB1-mCherry (data not shown).

### *ap-3*µ mutants show ABA hyposensitive phenotypes in seed germination and post-germination growth

To examine the physiological role of AP-3µ in plants, we obtained two different mutant lines, *ap-3µ-2* (SALK_064486C) and *ap-3µ-4* (CS859652), which carry T-DNA insertions in intron 5 and exon 8 of the *AP-3µ* gene, respectively (Supplementary Fig. S1A). Genomic PCR analyses verified that the T-DNA alleles were homozygous (Supplementary Fig. S1B). RT-PCR using the primer combinations PF2+PR2, confirmed the absence of full-length transcripts (Supplementary Fig. S1A and C).

**Fig. 3.**
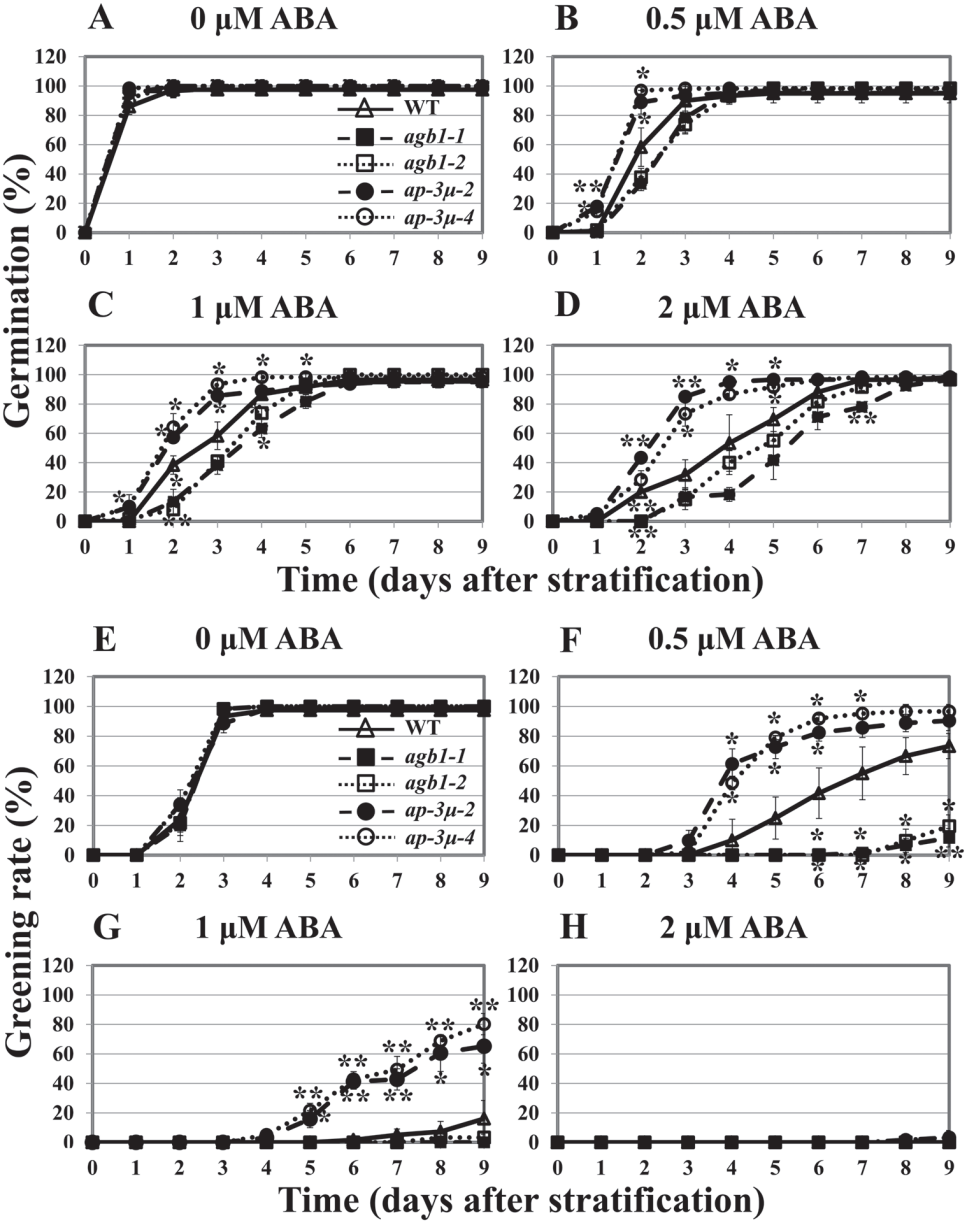
Seed germination and post-germination development of *ap-3µ* mutants are hyposensitive to ABA. (A–D) Germination rates of the wild-type (WT) seeds and *agb1-1*, *agb1-2*, *ap-3µ-2*, and *ap-3µ-4* mutant seeds in the presence of 0 (A), 0.5 (B), 1 (C), or 2 µM ABA (D). Germinated seeds were counted at the indicated time points. (E–H) Percentages of seedlings with fully expanded green cotyledons (greening rates) of WT and *agb1-1*, *agb1-2*, *ap-3µ-2*, and *ap-3µ-4* mutants in the presence of 0 (E), 0.5 (F), 1 (G), or 2 µM ABA (H). Seedlings with fully expanded green cotyledons were counted at the indicated time points. The experiment was repeated three times and data were averaged. *n*=20/genotype for each experiment. Error bars represent SD. *P<0.05, **P<0.005 as determined by t-test in comparison between wild type and each mutant.

In the presence of 0.5–2.0 µM ABA, *ap-3µ* seeds germinated earlier than did wild-type seeds (Fig. 3A–D). The effect of ABA on the post-germination growth of seedlings was analysed by determining the percentages of seedlings with fully expanded green cotyledons (greening rate) at 0.5–2.0 µM ABA. Greening rates of *ap-3µ* seedlings in the presence of 0.5 and 1.0 µM ABA were higher than those of wild-type seedlings (Fig. 3E–G and Supplementary Fig. S2). On the contrary, *agb1* mutants were hypersensitive to ABA during both germination and post-germination growth, as described previously (Pandey *et al.*, 2006). In the presence of 2.0 µM ABA, the wild type and each mutant line were able to germinate, but none of them formed green cotyledons (Fig. 3D and 3H). In the presence of ABA, which prevents the degradation of the seed storage proteins during germination (Garciarrubio *et al.*, 1997), the basic subunit of 12S globulin, which is a seed storage protein, degraded faster in *ap-3µ* mutant seedlings than in wild-type seedlings. In contrast, the basic subunit of 12S globulin was most preserved in *agb1* mutants (Supplementary Fig. S3). These results suggest that the *ap-3µ* mutants are less sensitive to ABA than the wild type. However, no difference between wild type and *ap-3µ-4* mutant was observed in the inhibition of root growth by ABA (Supplementary Fig. S4).

**Fig. 4.**
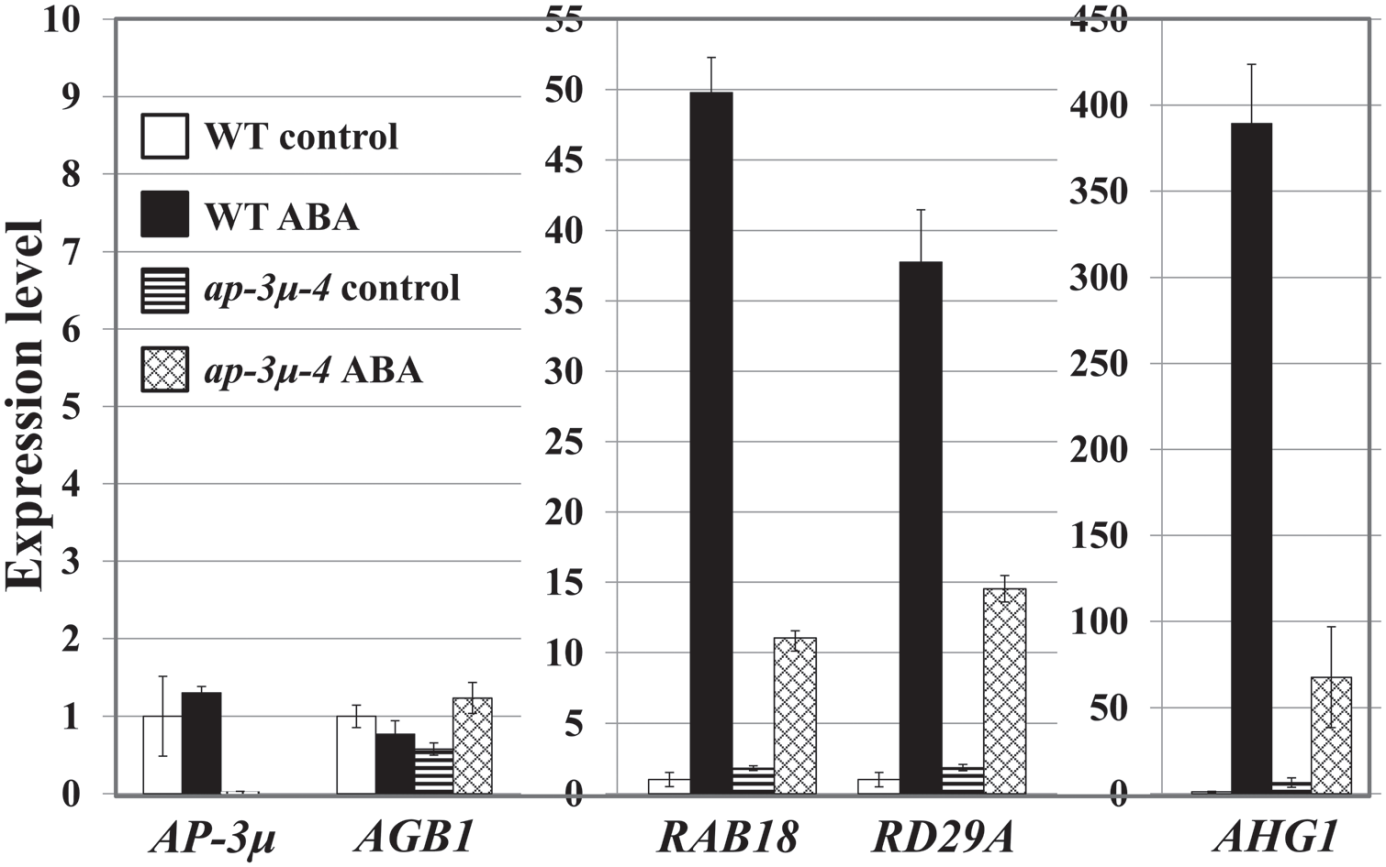
Expression of *AP-3µ*, *AGB1*, and ABA-responsive genes in wild type and *ap-3µ-4* mutant by real-time quantitative RT-PCR. The sample of wild type in the absence of ABA (WT control) was used as a reference sample. Relative expression levels were calculated by the ΔΔC_T_ method using *Actin* as an internal control gene. Experiments were performed in triplicate. Error bars represent SD. Wild type and *ap-3µ-4* mutant were grown on half-strength MS media with 0 (control) or 1.0 µM ABA for 18 days and used for cDNA synthesis for RT-PCR.

We investigated the expression profiles of *RAB18*, *RD29A*, and *AHG1*, which are ABA-induced marker genes. ABA-induced gene expression was reduced in *ap-3µ* mutants, as determined by the transcript levels of the marker genes (Fig. 4). No effect of ABA on expression of *AP-3µ* transcripts was observed. The expression of *AGB1* in the wild type did not change upon ABA treatment, while the expression of *AGB1* in *ap-3µ* mutant was upregulated and higher than that in the wild type in the presence of ABA (Fig. 4 left).

ABA also has roles in the responses to environmental stresses, including desiccation and high salinity (Busk and Pagès, 1998; Leung and Giraudat, 1998). However, when seeds and seedlings were exposed to various osmotic stresses (400mM mannitol, 150mM NaCl, or 9.2% polyethylene glycol), no difference was observed between the wild type and *ap-3µ* with respect to seed germination, seedling growth, or seedling development (Supplementary Figs. S5, S6, and S7). These data suggest that AP-3µ is not involved in the responses to either osmotic stress or salt stress.

### *Phenotypes of* agb1/ap-3µ *double mutants*

To investigate the interaction between *AP-3µ* and *AGB1* at the genetic level, we generated *agb1*/*ap-3µ* double mutants. A total of four double mutants were obtained; DM1-5-1, DM1-5-2, DM1-5-3, and DM2-8-5-5 (Supplementary Fig. S8). Because DM1-5-1, DM1-5-2, and DM1-5-3 are descended from the same line, DM1-5-3 and DM2-8-5-5 were selected for further analysis.

**Fig. 5.**
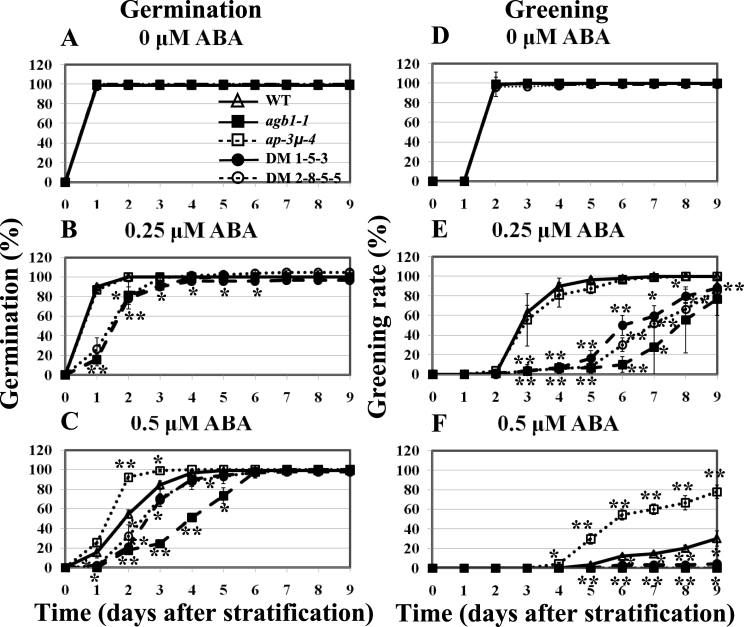
ABA sensitivity of *agb1*/*ap-3µ* double mutants during germination and post-germination growth. Germination rates (A–C) and greening rates (D–F) of wild type, *agb1-1*, *ap-3µ-4*, and *agb1*/*ap-3µ* double mutants in the presence of 0 (A and D), 0.25 (B and E), or 0.5 µM ABA (C and F) at the time indicated (days after stratification). The experiment was repeated three times and data were averaged. *n*=30/genotype for each experiment. Error bars represent SD. *P<0.05, **P<0.005 as determined by t-test in comparison between wild type and each mutant.

In the presence of 0.25 µM ABA, the germination rates of all the double mutants were similar to the germination rate of *agb1-1* mutant (Fig. 5B). In the presence of 0.5 µM ABA, the germination rates of all the double mutants were higher than the germination rate of the *agb1-1* mutant (Fig. 5C), suggesting that AP-3µ positively regulates the ABA response independently of AGB1 in seed germination. In the presence of 0.25 µM ABA, the greening rate of DM1-5-3 was significantly higher than the greening rate of *agb1-1* mutant only at day 6, while no significant difference was observed between DM2-8-5-5 and *agb1-1* mutant in their greening rates at any time points (Fig. 5E; see Supplementary Fig. S9E for t-test in comparison between *agb1-1* mutant and each genotypes). In the presence of 0.5 µM ABA, cotyledon greening was strongly inhibited in both the double mutants and *agb1-1* mutant (Fig. 5F; see Supplementary Fig. S10 for growth phenotypes in the presence of ABA). And the greening rate of DM1-5-3 was significantly but only slightly higher than the greening rate of *agb1-1* mutant at day 9, while no significant difference was observed between DM2-8-5-5 and *agb1-1* mutant in their greening rates at any time points (Supplementary Fig. S9F). These results suggest that the AP-3µ-dependent alleviation of the effects of ABA is at least partially dependent on AGB1 at the post germination stage.

Although *agb1* mutants have an increased number of lateral roots (Ullah *et al.*, 2003), the numbers of lateral roots were not significantly different between the wild type and *ap-3µ-4* mutant in the presence of 0 and 2 µM ABA. Similarly, the numbers of lateral roots were not different between *agb1-1* mutant and *agb1*/*ap-3µ* double mutants (Supplementary Fig. S11), suggesting that AP-3µ is not involved in regulating lateral root formation. Although lateral root formation can be controlled by auxin (Fukaki *et al.*, 2007 for review) and AGB1 is known to be involved in the auxin-dependent control of lateral root formation (Ullah *et al.*, 2003), the *ap-3µ* mutants and the wild type did not differ in their responses to an auxin, indole acetic acid, and an auxin-transport inhibitor, *N*-(1-naphthyl)phthalamic acid (data not shown). These results suggest that AP-3µ is not involved in the control of lateral root growth by auxin.

### Mutants of AP-3δ subunit and clathrin heavy chain (CHC) show ABA-hyposensitive phenotypes in post-germination growth

**Fig. 6.**
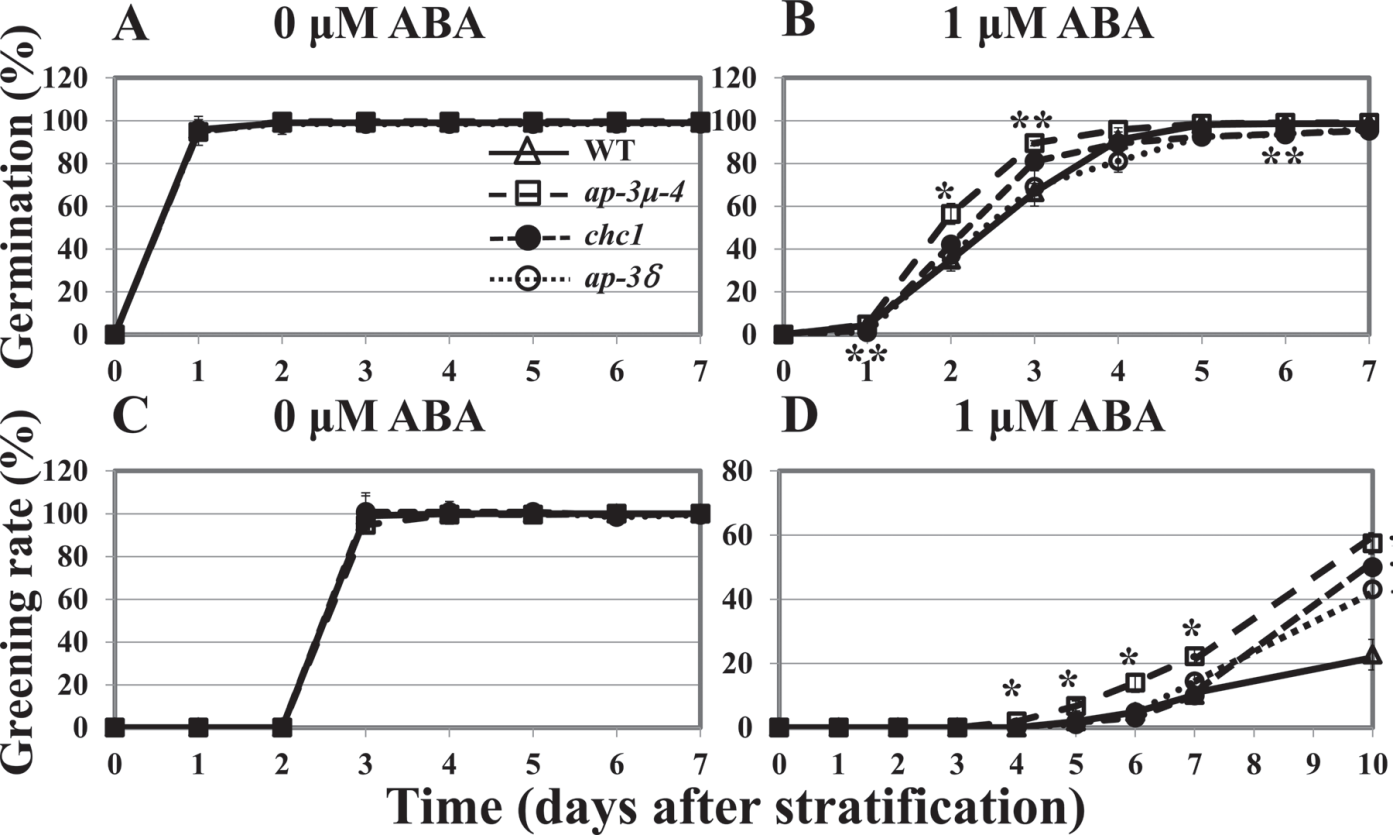
Mutants of AP-3δ subunit and Clathrin heavy chain show ABA-hyposensitive phenotype in post-germination growth. Germination rates (A and B) and greening rates (C and D) of wild type and *ap-3µ-4*, *ap-3δ*, and *chc1* mutants in the absence of ABA (A and C) or in the presence of 1.0 µM ABA (B and D) over time (days after stratification). The experiment was repeated three times for wild type and *ap-3µ-4* and *ap-3δ* mutants, and twice for *chc1* mutant. Data were averaged; *n*=70/genotype for each experiment. Error bars represent SD. *, *P*<0.05, **, *P*<0.005 as determined by t-test in comparison between wild type and each mutant.

The *ap-3δ* and *chc1* mutants harbour T-DNA insertions in exon 1 of the *AP-3δ* gene and exon 24 of the *CHC1* gene, respectively (Supplementary Fig. S12). Genomic PCR analyses confirmed that the T-DNA plants were homozygous (Supplementary Fig. S12). RT-PCR using primers specific for *AP-3*δ confirmed the absence of transcripts in *ap-3δ* (Supplementary Fig. S12A) and RT-PCR using primers specific for *CHC1* confirmed the absence of transcripts in *chc1* (Supplementary Fig. S12B). In the presence of 1.0 µM ABA, the rates of seed germination in *ap-3δ* and *chc1* were significantly but only slightly different from that in the wild type (Fig. 6B). However, in the greening test, only 23% of wild-type seedlings developed green cotyledons on day 10 at 1.0 µM ABA, whereas about 43% of the *ap-3δ* mutant seedlings and 50% of the *chc1* mutant seedlings developed green cotyledons (Fig. 6D). These results suggest that AP-3δ and CHC, as well as AP-3µ, function in the ABA response during post-germination growth.

## Discussion

### AP-3µ interacts with AGB1 and negatively regulates AGB1

**Fig. 7.**
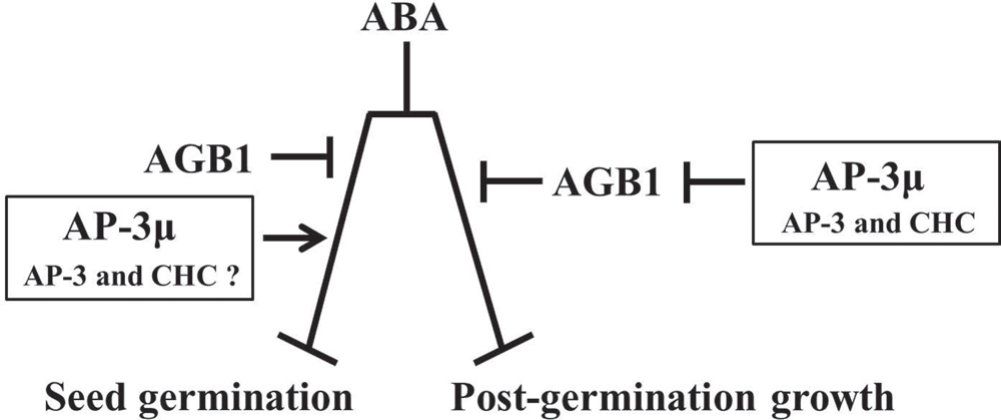
Schemes of AP-3µ modes of action. Contrary to AGB1, AP-3µ positively regulates the inhibition of seed germination and post-germination growth by ABA. AGB1 and AP-3µ function independently in ABA regulation of seed germination, but AP-3µ is a negative regulator of AGB1 in ABA regulation of post-germination growth. AP-3µ seems to function in these processes with other subunits of AP-3 complex mediating clathrin-based trafficking. AP-3, AP-3 complex; CHC, clathrin heavy chain.

We have shown that AP-3µ both physically and genetically interacts with AGB1 and regulates the ABA-dependent seed germination and cotyledon greening. Because AGB1 is a negative regulator of ABA responses (Pandey *et al.*, 2006), and because AP-3µ-dependent positive regulation of ABA responses during post-germination growth requires AGB1 (Fig. 5 and Supplementary Fig. S9), AP-3µ is thought to be an upstream negative regulator of AGB1 in the suppression of the inhibition of post-germination growth by ABA (Fig. 7). Although no information about the physical interaction between AGB1 and AP-3µ was available in *Arabidopsis* G-Signalling Interactome Database (AGIdb, http://bioinfolab.unl.edu/AGIdb), our results strongly support the idea that AP-3µ participates in the AGB1-mediated signalling.

Although ABA is known to be involved in acquiring tolerances to osmotic stress and salt stress, no difference was observed between the wild type and *ap-3µ* in osmotic stress or salt stress treatments (Supplementary Figs. S5, S6, and S7). These data suggest that AP-3µ is not involved in the responses to either osmotic stress or salt stress. Osmotic stresses can retard plant growth independently of ABA, because osmotic stresses inhibit cellular water uptake. In the case of salt stress, ion toxicity can also inhibit plant growth. It is possible that those ABA-independent plant growth inhibitions were much more significant than the ABA-mediated plant growth inhibition in our experiments in which the plants were subjected to osmotic/salt stresses.

In mammals, G-protein-coupled receptors are internalized to desensitize in response to excessive and/or continuous stimuli (Lefkowitz, 2004). An animal G-protein-coupled receptor, β2 adrenergic receptor, has been suggested to be internalized via clathrin-mediated endocytosis when it binds its ligand (Ferguson *et al.*, 1996; Schmid *et al.*, 2006; McMahon and Boucrot, 2011 for review). The classical function of clathrin-mediated endocytosis in the regulation of signal transduction is to terminate the signal by physically removing activated receptors from the cell surface (Sorkin and von Zastrow, 2009; Scita and Di Fiore, 2010). The internalization of ligand–receptor complexes into endosomes and then lysosomes may lead to their degradation, which results in termination of signalling. In plants, the internalization of AtRGS1 (regulator of G-protein signalling 1), which is the prototype of a seven-transmembrane receptor fused with an RGS domain, was reported (Urano *et al.*, 2012). AtRGS1 is known to be internalized when cells are treated with sugars such as d-glucose. Endocytosis of AtRGS1 physically uncouples the GTPase-accelerating activity of AtRGS1 from GPA1, permitting sustained activation of G-protein signalling on the plasma membrane (Urano *et al.*, 2013 for review). It is unclear whether the internalization of AtRGS1 is dependent on clathrin. Because AP-3µ is a component of a clathrin complex and interacts with AGB1, it will be interesting to examine whether AP-3µ is involved in the internalization of AtRGS1. Alternatively, it is possible that AGB1 is a direct target of the clathrin-mediated endocytosis. However, in either the presence or the absence of ABA, no difference was observed in the patterns of GFP-fused AGB1 (GFP-AGB1) signals between the wild type and *ap-3µ-4* mutant (Supplementary Fig. S13). It is possible that AP-3µ is involved in AGB1 internalization, but at least it could not be detected in this transient expression experiment. The level of AGB1, which negatively regulates ABA responses, might be higher in the absence of AP-3µ than in its presence, and this may be why the *ap-3µ* mutants showed hyposensitivities to ABA (Figs. 3 and 4). To our knowledge, this study is the first article reporting possibility of internalization of β subunit of G-protein in plants. However, further studies are required to elucidate whether AP-3µ is involved in endocytosis of AGB1 and other components of G-protein signalling.

The finding that the numbers of lateral roots were not significantly different between the wild type and *ap-3µ-4* mutant in either the absence or the presence of ABA (Supplementary Fig. S11), indole acetic acid, or *N*-(1-naphthyl)phthalamic acid (data not shown) suggests that AP-3µ does not function in regulating lateral root formation or in the control of lateral root growth by auxin. Therefore, the interaction between AP-3µ and AGB1 seems not to be involved in the control of lateral root formation and growth. In addition to the involvement in ABA-dependent inhibition of post-germination growth, the interaction between AP-3µ and AGB1 may be required in other processes. AGB1 mediates developmental processes and hormone responses. In addition to showing altered sensitivities to ABA and auxin, *agb1* mutants show altered sensitivities to gibberellin (Chen *et al.*, 2004), brassinosteroid (Chen *et al.*, 2004; Tsugama *et al.*, 2013), and jasmonic acid (Trusov *et al.*, 2006).

### AP-3 complex and clathrin are involved in ABA regulation of post-germination development

AP-3 exists in *Arabidopsis* as a complex (Zwiewka *et al.*, 2011). The CHC is also associated with AP-3β (Zwiewka *et al.*, 2011). AP-3β-GFP was found to localize predominantly in the cytoplasm (Feraru *et al.*, 2010). AP-3µ is present in the cytoplasm and nucleus (Fig. 2A). Each component of the AP-3 complex plays similar roles in regulating biogenesis and the functions of vacuoles in plants (Feraru *et al.*, 2010; Zwiewka *et al.*, 2011). Furthermore, *ap-3µ*, *ap-3δ*, and *ap-3β* all suppress the shoot gravitropism abnormality of the *zig1*/*vti11* mutant, which is defective in protein trafficking to the vacuoles (Sanmartín *et al.*, 2007; Niihama *et al.*, 2009). Similar phenotypes of the mutants defective in the different subunits of the same AP-3 complex suggest that these proteins act in the same process, possibly in the same complex. Also, the post-germination growth of the *ap-3µ*, *ap-3δ*, and *chc1* mutants were hyposensitive to ABA (Fig. 6D), supporting the idea that each subunit of AP-3 complex acts in the same process, probably mediating clathrin-based trafficking. However, the hyposensitivity to ABA during post-germination growth was greater in the *ap-3µ* mutants than in the *ap-3δ* and *chc1* mutants (Fig. 6D) and the rates of seed germination at 1 µM ABA in *ap-3δ* and *chc1* were significantly but only slightly different from that in the wild type (Fig. 6B). One possible explanation for these observations is that the homologue genes are redundant. The *Arabidopsis* genome encodes two *CHCs* that have 97% amino acid sequence identity (Kitakura *et al.*, 2011). The homologues of AP-3δ in other AP complexes may compensate for the loss of AP-3δ. Another possibility is that, although each subunit of the AP-3 complex acts in the same process in the ABA response during post-germination growth, AP-3µ is the predominant regulator in the process.

To our knowledge, this study is the first report on the involvement of AP-3 complex and clathrin in the regulation of post-germination growth by ABA. Further studies are needed to understand how the AP-3 complex and clathrin are involved in the ABA regulation of post-germination growth.

## Supplementary material

Supplementary data are available at *JXB* online.

Supplementary Method S1. Construction of pGAD-AP-3µ.

Supplementary Method S2. Constructions of pGEX-5X-AP-3µ and pGEX-5X- AP-3µ^DN^.

Supplementary Method S3. Induction and purification of GST-AP-3µ and GST-AP-3µ^DN^.

Supplementary Method S4. Construction of pBI121-35S-GFP, pBI121-35S-AP-3µ-GFP, pBI121-35S-mCherry, and pBI121-35S-AGB1-mCherry.

Supplementary Table S1. Primer pairs used for genomic PCR.

Supplementary Table S2. Primer pairs used for RT-PCR analyses.

Supplementary Fig. S1. Identification of *ap-3µ* T-DNA insertional mutants.

Supplementary Fig. S2. *ap-3µ* mutants are hyposensitive to ABA in post-germination growth.

Supplementary Fig. S3. The degradation of seed storage proteins occurs faster in *ap-3µ* mutants than in the wild type in the presence of ABA.

Supplementary Fig. S4. No difference between wild type and *ap-3µ-4* mutant was observed in the inhibition of root growth by ABA.

Supplementary Fig. S5. Responses of *ap-3µ* mutants to osmotic and salt stresses.

Supplementary Fig. S6. Germination rates of wild-type seeds and *agb1-1* and *ap-3µ-4* mutant seeds in the presence of 400mM mannitol or 9.2% polyethylene glycol.

Supplementary Fig. S7. Greening rates of wild type and *agb1-1* and *ap-3µ-4* mutants in the presence of 400mM mannitol or 9.2% polyethylene glycol.

Supplementary Fig. S8. Generation of *agb1*/*ap-3µ* double mutants.

Supplementary Fig. S9. T test for germination rates and greening rates in comparison between *agb1-1* mutant and each *agb1*/*ap-3µ* double mutants.

Supplementary Fig. S10. *agb1*/*ap-3µ* double mutants display ABA-hypersensitive phenotype in post-germination growth similar to that of *agb1* mutants.

Supplementary Fig. S11. Numbers of lateral roots of wild type, *agb1-1*, *ap-3µ-4*, and *agb1*/*ap-3µ* double mutants in the absence or in the presence of ABA.

Supplementary Fig. S12. T-DNA insertional mutants of AP-3δ and CHC1.

Supplementary Fig. S13. Subcellular localization of AGB1 in wild type and *ap-3µ* mutant.

Supplementary Data
