# Supplementary material for: The Arabidopsis adaptor protein AP-3µ interacts with the G-protein β subunit AGB1 and is involved in abscisic acid regulation of germination and post-germination development
Source: J Exp Bot. 2013 Oct 5;64(18):5611–21. doi: 10.1093/jxb/ert327 (PMC3871816; doi:10.1093/jxb/ert327)
Supplement: Supplementary Data [file supp_64_18_5611__index.html]

The Arabidopsis adaptor protein AP-3µ interacts with the G-protein β subunit AGB1 and is involved in abscisic acid regulation of germination and post-germination development — The Arabidopsis adaptor protein AP-3µ interacts with the G-protein β subunit AGB1 and is involved in abscisic acid regulation of germination and post-germination development — Supplementary Data 

# The *Arabidopsis* adaptor protein AP-3µ interacts with the G-protein β subunit AGB1 and is involved in abscisic acid regulation of germination and post-germination development

## Supplementary Data

Data files

**Files in this Data Supplement:**

- Supplementary Data - Supplementary Data
