# Supplementary material for: The Arabidopsis adaptor protein AP-3µ interacts with the G-protein β subunit AGB1 and is involved in abscisic acid regulation of germination and post-germination development
Source: J Exp Bot. 2013 Oct 5;64(18):5611–21. doi: 10.1093/jxb/ert327 (PMC3871816; doi:10.1093/jxb/ert327)
Supplement: Supplementary Data [file supp_ert327_jexbot106302_file001.pdf]

Title

The Arabidopsis adaptor protein AP-3μ interacts with the G-protein β subunit AGB1 and is involved in abscisic acid regulation of germination and post-germination development.

Names of the authors

Jeeraporn Kansup

Daisuke Tsugama

Shenkui Liu

Tetsuo Takano

Supplementary Method S1. Construction of pGAD-AP-3μ

A cDNA clone of *AP-3μ* (*AT1G56590*) (clone name: RAFL09-80-A11) was obtained from RIKEN BRC Experimental Plant Division (Seki *et al*., 2002). This clone had an 11-bp deletion (corresponding to position 247-257 from the start codon) in the putative full-length ORF of *AP-3μ* derived from a putative full-length cDNA clone of *AP-3μ* (GenBank accession: BX814222). To obtain the full-length ORF of *AP-3μ*, the 5’ region of *AP-3μ* ORF was amplified by PCR using the RIKEN cDNA clone as template and the following primer pair: 5’-CTCGGATCCATATGCTTCAATGTATCTTTCTC-3’ and 5’-GCAACTCTGCAAAGGAACTCGATTGCCATCAACGGTGGC-3’. The 3’ region of *AP-3μ* cDNA was amplified by PCR using the RIKEN cDNA clone as template and the following primer pair: 5’-TCGAGTTCCTTTGCAGAGTTGCTGATGTTTTGTCTGAG-3’ and 5’-CAGGAAACAGCTATGACCATGA-3’. The two kinds of PCR products (corresponding to the 5’ and 3’ regions of *AP-3μ* cDNA) were mixed and used as template for PCR using the following primer pair: 5’-CTCGGATCCATATGCTTCAATGTATCTTTCTC-3’ (*Bam*HI site is underlined) and 5’-CAGGAAACAGCTATGACCATGA-3’. The resultant PCR products contain the full-length ORF and the 3’ untranslated region (UTR) of *AP-3μ*. The PCR products were digested with *Bam*HI, and inserted into the *Bgl*II-*Sma*I site of pGADT7-Rec (Clontech, Japan), generating pGAD-AP-3μ.

Reference:

Seki M, Narusaka M, Kamiya A, et al. 2002. Functional annotation of a full-length Arabidopsis cDNA collection. Science 296, 141-145.

Supplementary Method S2. Constructions of pGEX-5X-AP-3μ and pGEX-5X- AP-3μ^{DN}

To express GST-fused AP-3μ (GST-AP-3μ), the open reading frame (ORF) of *AP-3μ* was amplified by PCR using pGAD-AP-3μ as template and the following primer pair: 5′-CGCGGGATCCGGATGCTTCAATGTATCTTTCTCATCTCCGAT-3′ and 5′-GGCGCCCGGGTACAACCTGACATCGAACTCACCAGC-3′ (*BamH*I and *Sma*I sites are underlined). The PCR products were digested by *BamH*I and *Sma*I, and cloned into the *BamH*I-*Sma*I site of pGEX-5X-1 (GE Healthcare, UK), generating pGEX-5X-AP-3μ.

To express GST-fused AP-3μ^{DN} (GST-AP-3μ^{DN}), the open reading frame (ORF) of *AP-3μ*^{DN} was amplified by PCR using pGAD-AP-3μ as template and the following primer pair: 5′-AATTCCCGGGAATGCTTCAATGTATCTTTCT -3′ and 5′-AATTGGATCCACAAACGAGGAGGGATAGTTTGAAG-3′ (*Xma*I and *BamH*I sites are underlined). The PCR products were digested by *Xma*I and *BamH*I, and cloned into the *Xma*I-*BamH*I site of pGBKT7 (Clontech, Japan), generating pGBK-AP-3μ^{DN}. pGBK-AP-3μ^{DN} was digested by *Sma*I and *Not*I, and the resultant ORF fragments of AP-3μ^{DN} were inserted into the *Sma*I-*Not*I site of pGEX-5X-1 (GE Healthcare, UK) in-frame to the coding sequence of glutathione S-transferase (GST), generating pGEX-5X- AP-3μ^{DN}.

Supplementary Method S3. Induction and purification of GST-AP-3μ and GST-AP-3μ^{DN}

To induce GST-AP-3μ or GST-AP-3μ^{DN}, pGEX-5X-AP-3μ or pGEX-5X-AP-3μ^{DN} was transformed into the *Escherichia coli* strain, BL21 (DE3). The transformed *E. coli* cells were cultured at 37°C in LB medium until OD_{600} reached 0.5, and incubated at 28 °C for 3 h after an addition of IPTG to a final concentration of 0.1 mM. The cells were then harvested by centrifugation and resuspended in 1× PBS (phosphate-buffered saline: 137 mM NaCl, 8.10 mM $Na_2HPO_4 \cdot 12H_2O$, 2.68 mM KCl, 1.47 mM KH_2PO_4, pH 7.4) with 2 mg/ml lysozyme (Wako, Japan). The cell suspension was frozen at −80 °C and thawed at room temperature. Freezing and thawing were repeated two more times to lyse the cells, and 2 units of recombinant DNase I (Takara, Japan) was added to the solution. The solution was incubated at room temperature until the solution became fluid due to DNA degradation. The solution was then centrifuged at 12000 ×g for 5 min and the supernatant was used as crude protein extracts.

Supplementary Method S4. Constructions of pBI121-35S-GFP, pBI121-35S-AP-3μ-GFP, pBI121-35S-mCherry and pBI121-35S-AGB1-mCherry

To create plasmids for expressing GFP-fused protein, pBS-35SMCS-GFP (Tsugama *et al*., 2012a) was digested by *Sma*I and *EcoR*I to obtain the DNA fragment containing GFP-Nos terminator. This fragment was inserted into the *Sma*I-*EcoR*I site of a pre-digested pBI121, which GUS-Nos terminator fragment was removed, generating pBI121-35S-GFP.

The open reading frame (ORF) of *AP-3μ* was amplified by PCR using pGAD-AP-3μ as template and the following primer pair: 5′-CCGGTCTAGAATGCTTCAATGTATCTTTCTC-3′ and 5′-GGCGCCCGGGTACAACCTGACATCGAACTCACCAGC-3′ (*Xba*I and *Sma*I sites are underlined). The PCR products were digested by *Xba*I and *Sma*I, and cloned into the *Xba*I-*Sma*I site of pBI121-35S-GFP, generating pBI121-35S-AP-3μ-GFP.

To create plasmids for expressing mCherry-fused protein, the ORF of mCherry was amplified by PCR using pmCherry-N1 (Clontech, Japan) as template and the following primer pair: 5′- ATATCCCGGGTATGGTGAGCAAGGGCGAGGAGGATAACATG-3′ and 5′-AATTCCCGGGTTACTTGTACAGCTCGTCCATGCCGCCGGT-3′ (*Sma*I site is underlined). The PCR fragments were digested by *Sma*I and inserted into the *Sma*I site of pBI121, generating pBI121-35S-mCherry. The open reading frame (ORF) of *AGB1* was amplified by PCR using pGBK-AGB1 (Tsugama *et al*., 2012b) as template and the following primer pair: 5′-GGCCTCTAGAATGTCTGTCTCCGAGCTCAAAGAACGCCAC-3′ and 5′-TTAAGGATCCAATCACTCTCCTGTGTCCTCCAAACGCCCAT -3′ (*Xba*I and *BamH*I sites are underlined). The PCR products were digested by *Xba*I and *BamH*I, and cloned into the *Xba*I-*BamH*I site of pBI121-35S-mCherry, generating pBI121-35S-AGB1-mCherry.

Reference:

Tsugama D, Liu S, Takano T. 2012a. A putative myristoylated 2C-type protein phosphatase, PP2C74, interacts with SnRK1 in Arabidopsis. FEBS Letters 586, 693-698.

Tsugama D, Liu H, Liu S, Takano T. 2012b. Arabidopsis heterotrimeric G protein β subunit interacts with a plasma membrane 2C-type protein phosphatase, PP2C52. Biochimica et Biophysica Acta 1823, 2254-2260.

Supplementary Table S1. Primer pairs used for genomic PCR

Gene	Primer sequence (5' > 3')
AP-3μ	PF1: CCGGTCTAGAATGCTTCAATGTATCTTTCTC<br>PR1: AATTGGATCCACAAACGAGGAGGGATAGTTTGAAG
AP-3δ	Fw: GGGCACTGCTCATTGAT<br>Rv: GTGGTTAATGCTGGTC
CHC1	Fw: GGTGGATAGTGAGCTCATCT<br>Rv: CTTTCTCGGGAACTAGGATCTG

Supplementary Table S2. Primer pairs used for RT-PCR analyses

Gene	Primer sequence (5' > 3')
AP-3μ	PF2: CCCGTGTGTCCGCTACAGACC<br>PR2: TGGGATTCGCCCGATTGTCCA
UBQ5	Fw: GACGCTTCATCTCGTCC<br>Rv: CCACAGGTTGCGTTAG
RAB18	Fw: TCCAGCTCTAGCTCGGAGGATGA<br>Rv: GGATCCCATGCCGCCCATCG
RD29A	Fw: GCCGGAATCTGACGGCGGTT<br>Rv: CCCGTCGGCACATCCTTGTCG
AHG1	Fw: AGGATGGATGAGATGGCAAC<br>Rv: CCTCCCTCGGATCACAGTTA
Actin	Fw: GGTAACATTGTGCTCAGTGGTGG<br>Rv: AACGACCTTAATCTTCATGCTGC
AGB1	Fw: TGGGATGTAACTACTGGTCTCA<br>Rv: CAGCACGAGTGTCCCACAA
AP-3δ	Fw2: GAGATCCGACGAAACATATTTCTAA<br>Rv2: TGCTGGTCTTATCTTCGTCACT
CHC1	Fw: GGTGGATAGTGAGCTCATCT<br>Rv: CTTTCTCGGGAACTAGGATCTG

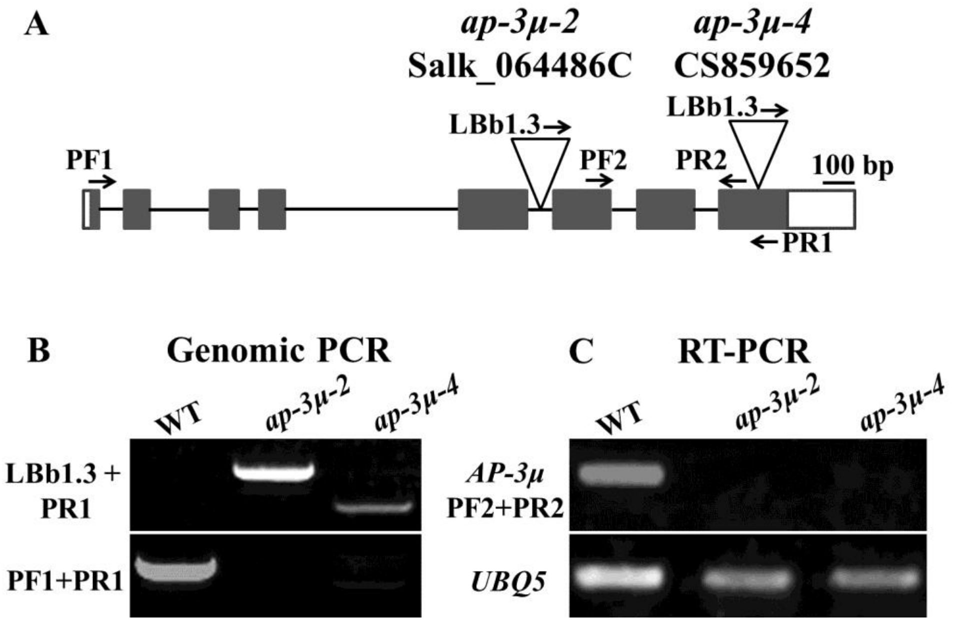

Supplementary Figure S1. T-DNA insertional mutants of AP-3μ.

(A) Schematic representation of the *AP-3μ* gene. Gray boxes and white boxes represent exons and UTR regions, respectively. The positions of the *ap-3μ-2* and *ap-3μ-4* T-DNA insertions are indicated. Primer pairs used in genomic PCR screening and primer pairs used in RT-PCR to assess *AP-3μ* transcripts are indicated. (B) Genomic PCR analyses verified homozygosity for the T-DNA alleles (C) RT-PCR analysis of *AP-3μ* transcript levels in *ap-3μ* mutants compared with wild-type. *UBQ5* was used as an internal control.

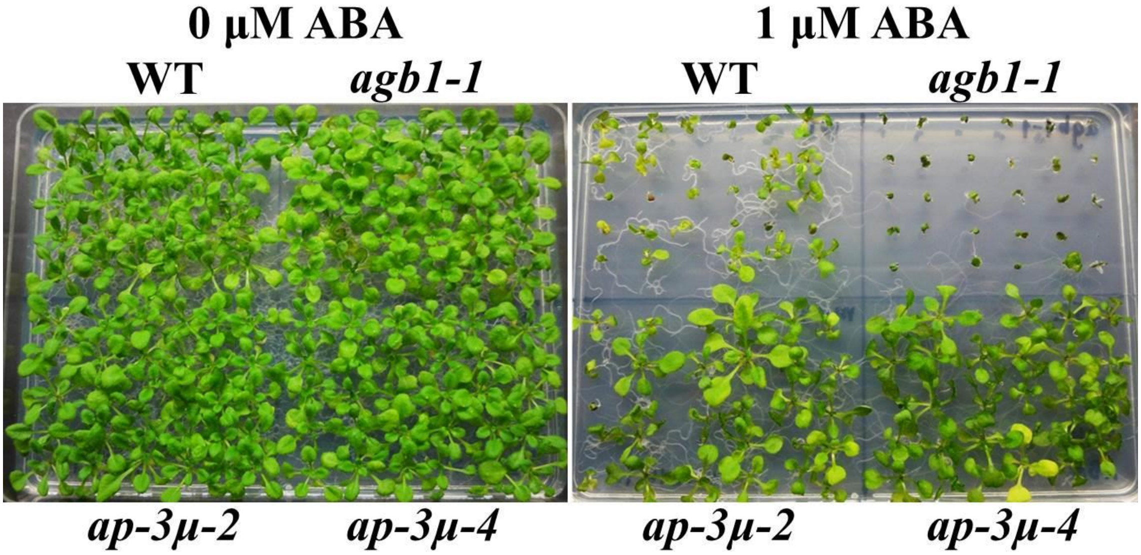

Supplementary Figure S2. *ap-3μ* mutants are hyposensitive to ABA in post-germination growth. Plants were grown on the media containing 0 and 1 μM ABA. Photographs were taken 20 days after germination.

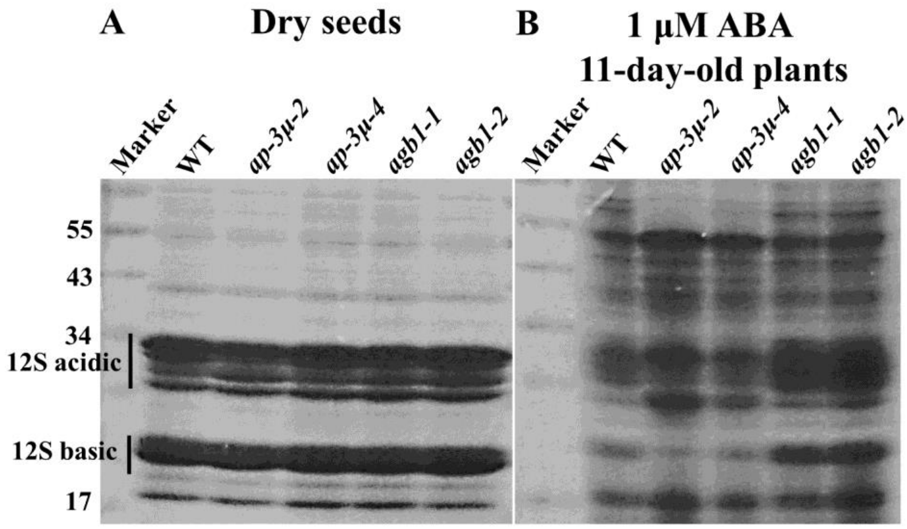

Supplementary Figure S3. The degradation of seed storage proteins occurs faster in *ap-3µ* mutants than in the wild-type in the presence of ABA. Total proteins were extracted from dry seeds (A) and 11-day-old plants grown in the presence of 1 µM ABA (B), and separated by SDS-PAGE. The Arabidopsis storage protein clusters are indicated (acidic and basic subunits of 12S globulin). Experiments were performed 3 times and a representative result is shown.

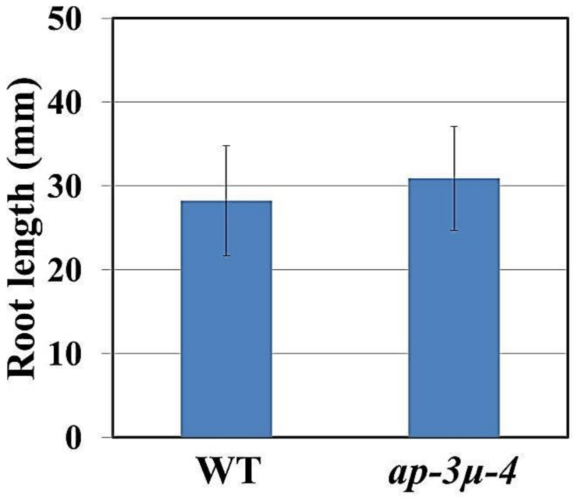

Supplementary Figure S4. No difference between wild-type and *ap-3μ-4* mutant was observed in the inhibition of root growth by ABA. Wild-type and *ap-3μ-4* mutant seedlings were grown vertically on half-strength MS media without ABA for 3 days, and then transferred to half-strength MS media with 20 μM ABA and grown vertically for 10 days. The error bars represent SD. n = 24.

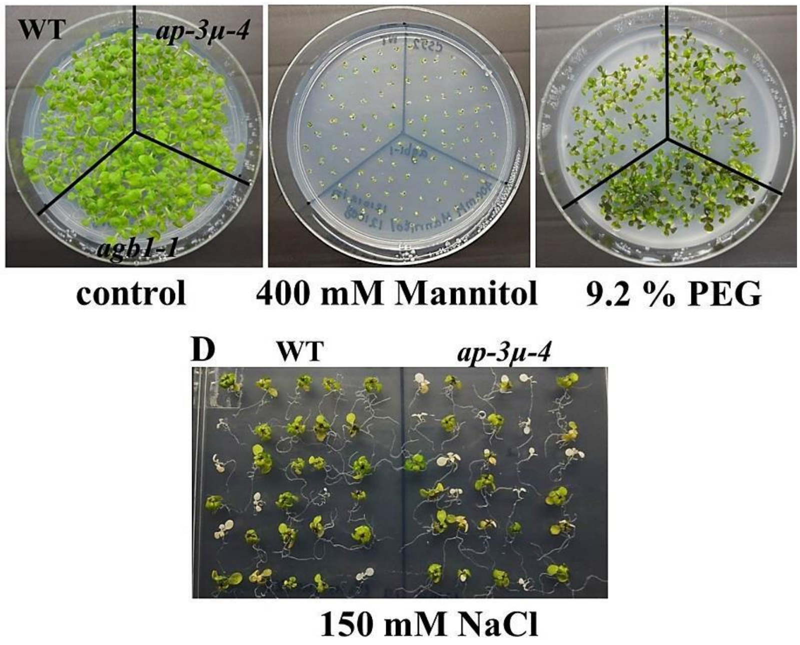

Supplementary Figure S5. Responses of ap-3mu mutants to osmotic and salt stresses.

Wild-type and *ap-3μ-4* and *agb1-1* mutant seedlings were grown on half-strength MS media without treatment (A, control) or with 400 mM mannitol (B) or with 9.2% polyethylene glycol (PEG) (C). Photographs were taken on day 13 after transfer of seeds to light at 22˚C.

(D) Wild-type and *ap-3μ-4* mutant seedlings were grown on half-strength MS media without treatment for 6 days, transferred to half-strength MS media with 150 mM NaCl and further grown. Photographs were taken 11 days after plants were transferred to the NaCl media.

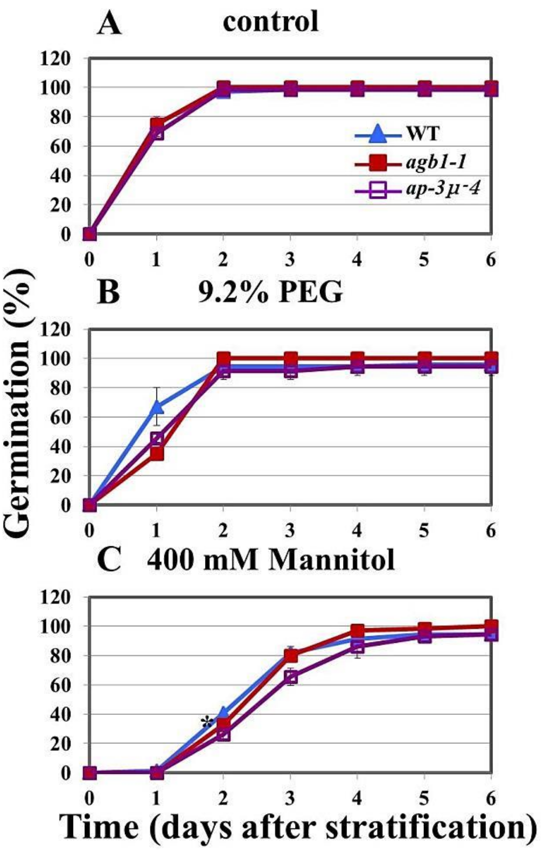

Supplementary Figure S6. Germination rates of wild-type seeds and *agb1-1* and *ap-3μ-4* mutant seeds in the presence of 400 mM mannitol or 9.2% polyethylene glycol.

Germination rates of wild-type seeds and *agb1-1* and *ap-3μ-4* mutant seeds in control media (A), in the presence of 400 mM mannitol (B) or 9.2% polyethylene glycol (C) over time (days after stratification). The experiment was repeated three times and data were averaged. n = 35/genotype for each experiment. The error bars represent SD. *, $p < 0.05$, **, $p < 0.005$ as determined by t test in comparison between wild-type and each mutant.

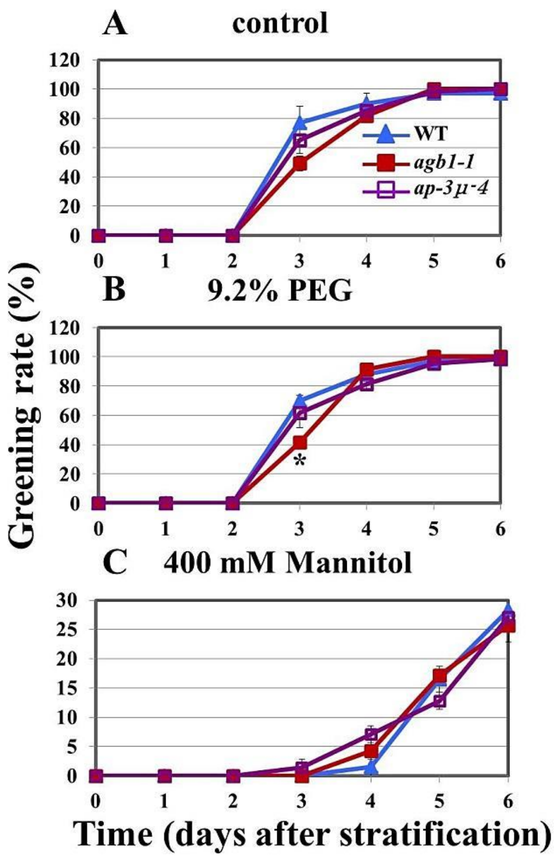

Supplementary Figure S7. Greening rates of wild-type and *agb1-1* and *ap-3μ-4* mutants in the presence of 400 mM mannitol or 9.2% polyethylene glycol.

Greening rates of wild-type and *agb1-1* and *ap-3μ-4* mutants in control media (A), in the presence of 400 mM mannitol (B) or 9.2% polyethylene glycol (C) over time (days after stratification). The experiment was repeated three times and data were averaged. n = 35/genotype for each experiment. The error bars represent SD. *, $p < 0.05$, **, $p < 0.005$ as determined by t test in comparison between wild-type and each mutant.

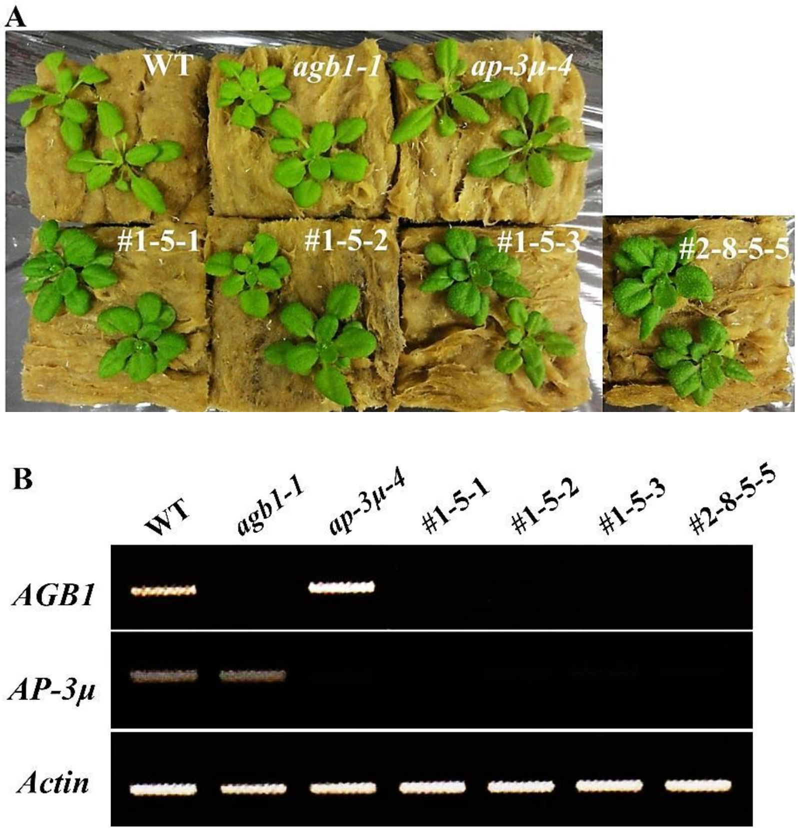

Supplementary Figure S8. Generation of *agb1/ap-3μ* double mutants.

(A) Total of four double mutant lines (DM) were generated; DM#1-5-1, DM#1-5-2, DM#1-5-3, and DM#2-8-5-5. Wild-type, *agb1-1*, *ap-3μ-4*, and *agb1/ap-3μ* double mutants were grown on half-strength MS media plates containing 1% Suc for 10 days and transferred onto rockwool cubes and grown further with 0.2× MS solution regularly supplied. The photographs of three-week-old plants were taken.

(B) RT-PCR using the primers specific to *AGB1* or *AP-3μ* gene confirmed the absence of transcripts of *AGB1* or *AP-3μ*. *Actin* was used as a reference transcript.

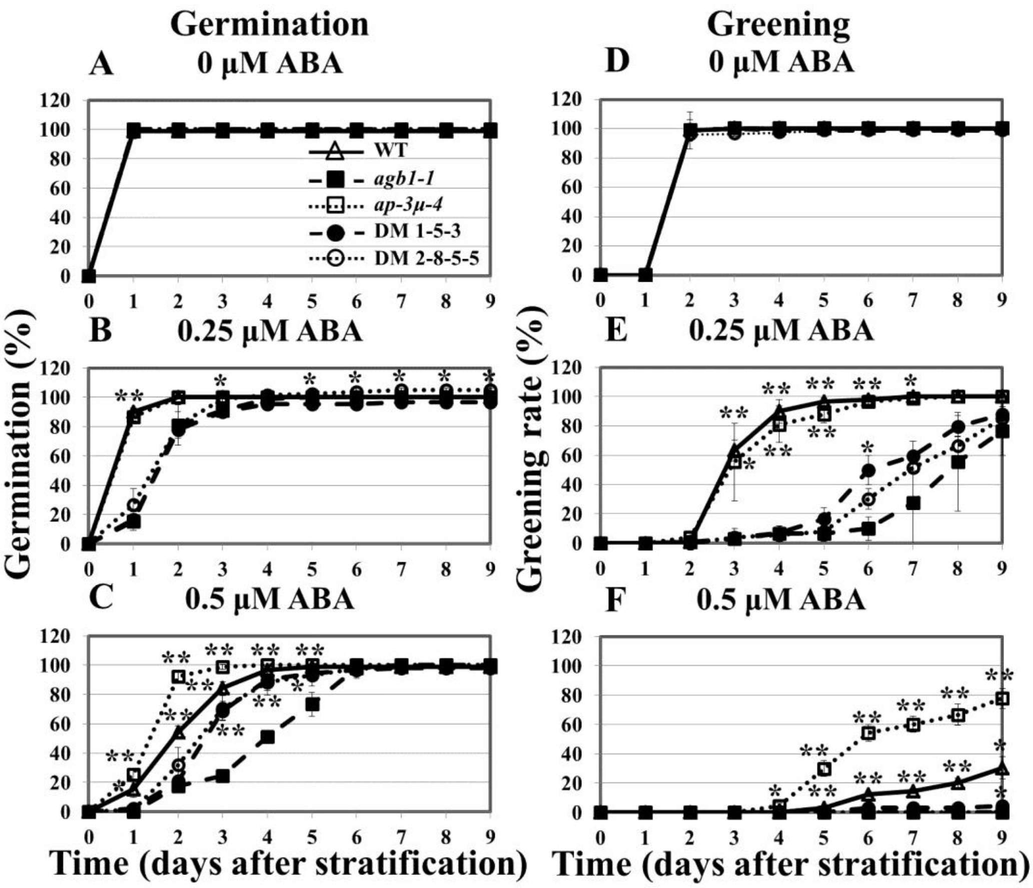

Supplementary Figure S9. T test for germination rates and greening rates in comparison between *agb1-1* mutant and each *agb1/ap-3μ* double mutants.

Germination rates (A to C) and greening rates (D to F) of wild-type, *agb1-1*, *ap-3μ-4*, and *agb1/ap-3μ* double mutants in the presence of 0 (A and D), 0.25 (B and E) or 0.5 μM ABA (C and F) at the time indicated (days after stratification). The experiment was repeated three times and data were averaged. n = 30/genotype for each experiment. The error bars represent SD. *, $p < 0.05$, **, $p < 0.005$ as determined by t test in comparison between *agb1-1* mutant and each genotypes.

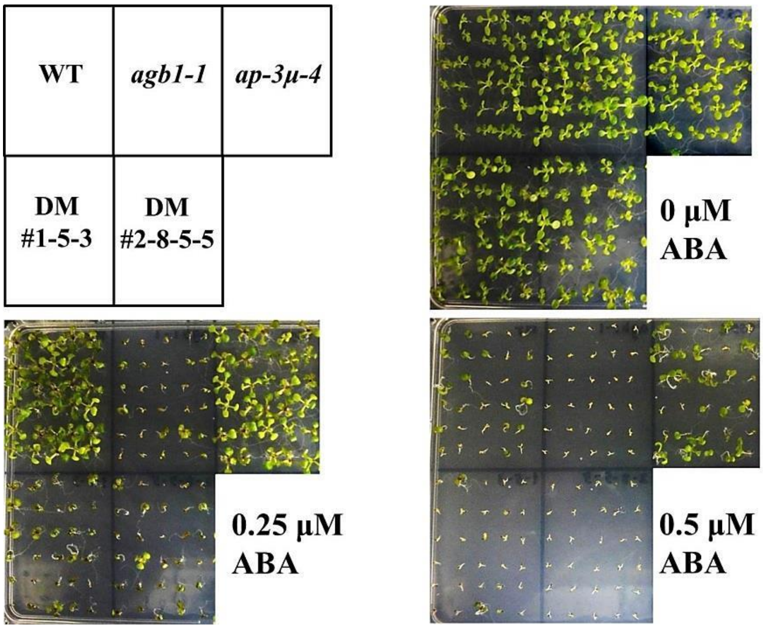

Supplementary Figure S10. ***agb1/ap-3µ*** **double mutants display ABA-hypersensitive phenotype in post-germination growth similar to that of** ***agb1*** **mutants.** Plants were grown in the presence of 0, 0.25 or 0.5 µM ABA. Photographs were taken on day 9.

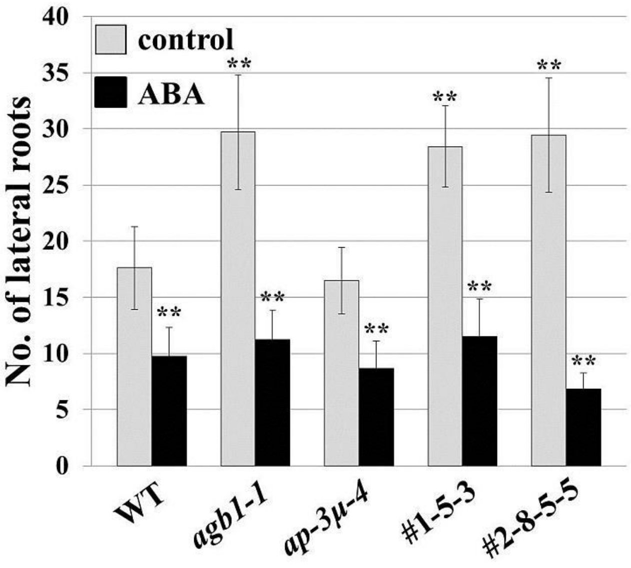

Supplementary Figure S11. Numbers of lateral roots of wild-type, *agb1-1*, *ap-3μ-4,* and *agb1/ap-3μ* double mutants in the absence or in the presence of ABA.

Seeds germinated on 0.5× MS plates were transferred after 3 d to control plates (no ABA) or plates containing 2 μM ABA and seedlings were allowed to grow vertically for 12 d under 16-h light/8-h dark condition. Number of lateral roots was counted. Figure shows number of lateral roots in different genotypes in presence or absence of ABA. The error bars represent SD. n = 9-12. *, $p < 0.05$, **, $p < 0.005$ as determined by t test in comparison with wild-type control.

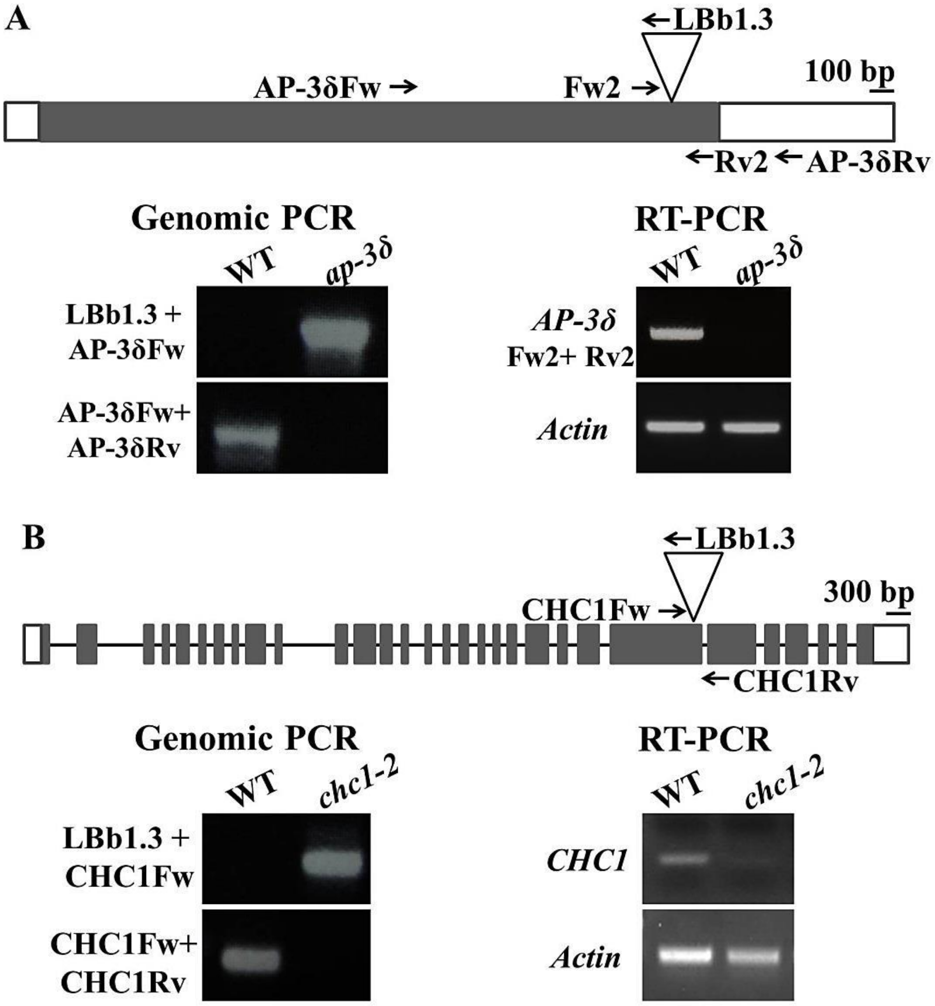

Supplementary Figure S12. T-DNA insertional mutants of AP-3δ and CHC1.

Schematic representations of the *AP-3δ* (A) and *CHC1* gene (B). Gray boxes and white boxes represent exons and UTR regions, respectively. The positions of the T-DNA insertions are indicated. Primer pairs used in genomic PCR are indicated. Genomic PCR analyses verified homozygosity of each mutants (left panels). Gene-specific primers used in RT-PCR to confirm absence of full-length transcripts in mutants (right panels). *Actin* was used as a reference transcript.

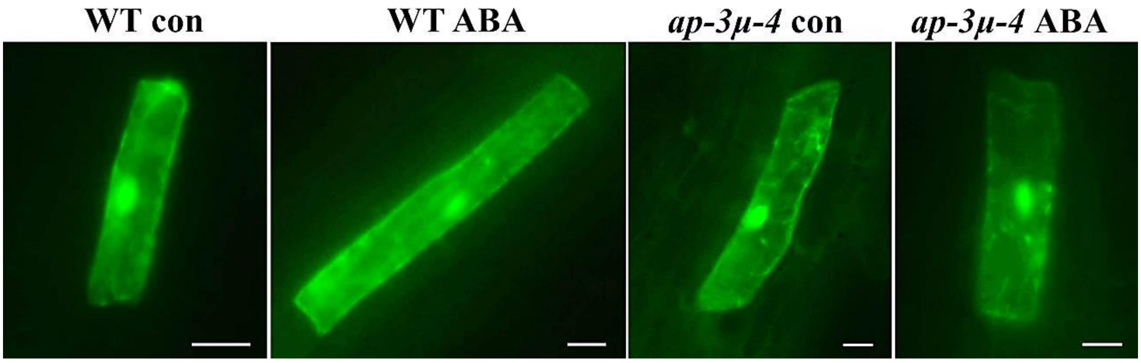

Supplementary Figure S13. Subcellular localization of AGB1 in wild-type and *ap-3μ* mutant. Wild-type and *ap-3μ* mutant were grown on 0.5× MS plates for 10 d and transferred to control plates (no ABA, con) or plates containing 1 μM ABA for 24 h and seedlings were used for transient expression of pBS-35S-GFP-AGB1 (Tsugama *et al*., 2012). More than 10 cells were observed and a representative cell is shown. Scale bars = 20 μm.

Reference:

Tsugama D, Liu H, Liu S, Takano T. 2012. Arabidopsis heterotrimeric G protein β subunit interacts with a plasma membrane 2C-type protein phosphatase, PP2C52. Biochimica et Biophysica Acta 1823, 2254-2260.
